# Revisiting Textbook Azide-Clock Reactions: A “Propeller-Crawling” Mechanism Explains Differences in Rates

**DOI:** 10.1021/jacs.4c03360

**Published:** 2024-04-30

**Authors:** Anthony T. Bogetti, Matthew C. Zwier, Lillian T. Chong

**Affiliations:** † Department of Chemistry, University of Pittsburgh, Pittsburgh, Pennsylvania 15260, United States; ‡ Department of Chemistry, Drake University, Des Moines, Iowa 50311, United States

## Abstract

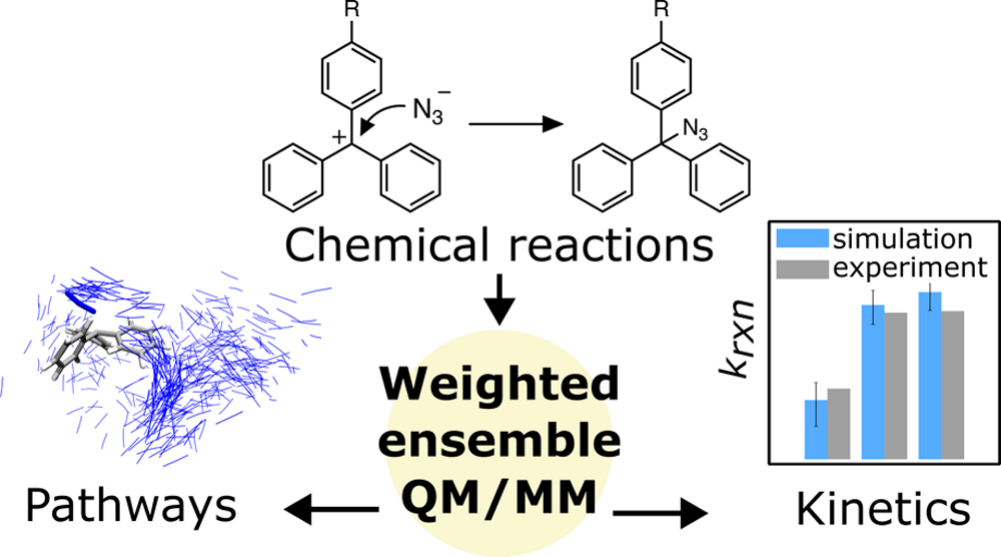

An ongoing challenge to chemists is the analysis of pathways and kinetics for chemical reactions in solution, including transient structures between the reactants and products that are difficult to resolve using laboratory experiments. Here, we enabled direct molecular dynamics simulations of a textbook series of chemical reactions on the hundreds of ns to μs time scale using the weighted ensemble (WE) path sampling strategy with hybrid quantum mechanical/molecular mechanical (QM/MM) models. We focused on azide-clock reactions involving addition of an azide anion to each of three long-lived trityl cations in an acetonitrile–water solvent mixture. Results reveal a two-step mechanism: (1) diffusional collision of reactants to form an ion-pair intermediate; (2) “activation” or rearrangement of the intermediate to the product. Our simulations yield not only reaction rates that are within error of experiment but also rates for individual steps, indicating the activation step as rate-limiting for all three cations. Further, the trend in reaction rates is due to dynamical effects, i.e., differing extents of the azide anion “crawling” along the cation’s phenyl-ring “propellers” during the activation step. Our study demonstrates the power of analyzing pathways and kinetics to gain insights on reaction mechanisms, underscoring the value of including WE and other related path sampling strategies in the modern toolbox for chemists.

## Introduction

Dynamical effects on chemical reactions in solution—involving the atomic motions and associated kinetics—have been increasingly recognized as important features of reaction mechanisms.^1−3^ Of great interest is therefore the generation of complete atomically detailed pathways from the reactants to the products. While spectroscopy experiments in solvent environments can detect product formation and measure the rate constant for the overall reaction, such experiments are unable to directly provide rate constants of individual steps or atomic structures of transient states. In principle, molecular dynamics simulations can complement experiment by providing direct estimates of rate constants for individual steps and generate complete reaction pathways at femtosecond resolution, including transient states that are too fleeting to be captured by experiments. However, the use of either fully quantum mechanical, ab initio molecular dynamics (AIMD), or hybrid quantum mechanical/molecular mechanics (QM/MM) simulations is required for the modeling of chemical reactions, and these simulations are, in general, computationally prohibitive for the goals of generating pathways and estimating rates. These goals have also been elusive to enhanced sampling methods that modify the free energy landscape (e.g., ab initio nanoreactor;^4^ metadynamics^5^ with AIMD^6−8^ or hybrid QM/MM models^9^), as such methods efficiently provide thermodynamics observables and predictions of products, but at the expense of pathways with rigorous kinetics.

One promising class of enhanced sampling methods that maintains rigorous kinetics is path sampling (e.g., transition path sampling^10,11^ and weighted ensemble sampling^12,13^). Path sampling approaches focus computing power on the functional transitions between stable states rather than the stable states themselves,^14^ exploiting the fact that the transition time over the effective free energy barrier can be orders of magnitude faster than the waiting time in the initial stable state. For example, transition path sampling has been used to generate pathways for enzyme-catalyzed reactions^15,16^ and pathways along with rate constants for both chemical reactions in solution^17,18^ and enzyme-catalyzed reactions.^19^ While weighted ensemble path sampling has not yet been applied to simulations of chemical reactions—until now—the strategy has been demonstrated to be orders of magnitude more efficient than conventional simulations (without enhanced sampling) in generating pathways and/or rate constants for complex processes that range from microseconds (e.g., binding processes of proteins^20,21^ and DNA^22^) to milliseconds (e.g., protein folding^23^) to seconds (e.g., large-scale conformational switching in proteins^24,25^) and beyond (e.g., protein–ligand unbinding^26^).

**Figure 1:**
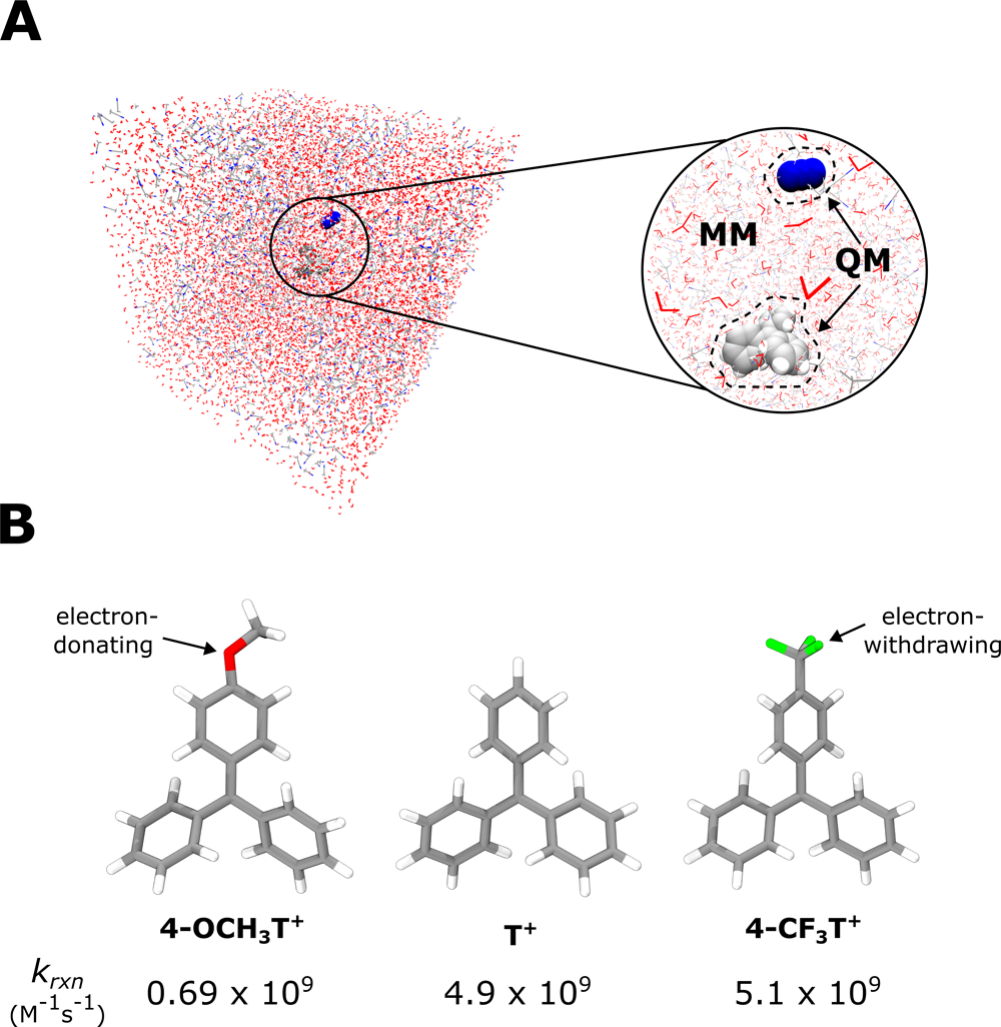
Azide-clock reactions in this study involved the addition of an azide anion to three different trityl cations with reaction rate constants *k*_rxn_ that span an order of magnitude. (A) A cubic box containing the reactant solutes and explicit 1:2 v/v acetonitrile–water solute mixture, with the solutes modeled quantum mechanically and the solvent represented using classical molecular mechanics models. (B) The three cations are shown in order of increasing reaction rate constant *k*_rxn_ for addition of an azide anion. The 4-OCH_3_T^+^ cation contains an electron-donating methoxy substituent, and the 4-CF_3_T^+^ cation contains an electron-withdrawing trifluoro substituent. The *k*_rxn_ values shown are published from previous laser flash photolysis experiments by others.^27^

Here we applied the weighted ensemble (WE) strategy to enable hybrid QM/MM simulations of textbook azide-clock reactions in a 1:2 v/v acetonitrile–water mixture of explicit solvent molecules (Figure 1A). Each of these reactions involves addition of an azide anion to a long-lived cation, and the reactions are generally assumed to occur at the diffusion limit (5 × 10^9^ M^–1^ s^–1^)^27^ such that the azide functions as a “clock” for the lifetimes of the cations.^27,28^ We simulated azide addition to each of three “propeller-shaped” trityl cations (Figure 1B): the unsubstituted cation (T^+^), a cation with an electron-donating methoxy substituent (4-OCH_3_T^+^), and a cation with an electron-withdrawing trifluoromethyl substituent (4-CF_3_T^+^). As measured by laser flash photolysis, the rate constants for these reactions span an order of magnitude, ranging in time scale from hundreds of ns to μs.^27^ Our simulations not only yielded ensembles of reaction pathways but also direct calculations of rate constants.

## Methods

### Preparation of the Hybrid QM/MM Model

For each reaction simulation, we used a hybrid QM/MM model in which the QM region consisted of the reactants (azide anion and trityl cation) and the MM region consisted of the solvent molecules (1:2 v/v acetonitrile–water mixture). The QM region was modeled using the PM6-D semiempirical method^29^ and contained 41 atoms for the 4-OCH_3_T^+^ reaction, 37 atoms for the T^+^ reaction, and 40 atoms for the 4-CF_3_T^+^ reaction. We benchmarked higher levels of QM theory such as the RI-MP2/cc-pVTZ and B3LYP/6-31G levels, but the single-point energy calculations were too computationally expensive for dynamics propagation in our WE simulations (0.24 ps/day for RI-MP2 and 1.85 ps/day for B3LYP vs 820 ps/day for PM6-D). van der Waals parameters from the GAFF force field were used for the azide and cations.^30^ The MM region was presented using TIP3P water molecules^31^ and acetonitrile molecules using compatible parameters.^32^ Interaction energies between the QM and MM regions were treated with electrostatic embedding and long-range electrostatics with the particle mesh Ewald method.^33^

We generated starting models separately for the azide anion, the three trityl cations, and solvent molecules (acetonitrile and water) by (i) constructing the molecular models separately and energy-minimizing the models in vacuum using the Avogadro software and the GAFF force field^30^ and (ii) optimizing the geometry of each model at the RI-MP2 level of theory^34^ with the cc-pVTZ basis set and a cc-pVDZ/C auxiliary basis set using the Orca 4.1.2 software package (see Figure S1 for the optimized geometries).^35^ For each of the three cations, we positioned the geometry optimized cation and azide conformations at a 20 Å separation distance between the central carbon of the cation and the nearest azide nitrogen along a vector perpendicular to the plane of the cation. Next, we used the PACKMOL package^36^ to solvate the reactants with a pre-equilibrated cubic box containing a 1:2 v/v acetonitrile–water mixture of the corresponding geometry-optimized molecules and a 20 Å buffer distance between the reactants and the edges of the box. The pre-equilibrated solvent box was previously subjected to energy minimization and 20 ps of NVT equilibration followed by 1 ns of NPT equilibration using the AMBER 18 software package.^37^

### Simulation Workflow

Our workflow for simulating the chemical reactions involved two stages: (1) conventional simulations of the unassociated reactants; (2) weighted ensemble simulations of the chemical reaction starting from configurations sampled in stage #1. Full details are provided below.

### Conventional Simulations of the Reactants

The solvated starting model of each pair of unassociated reactants was energy minimized and equilibrated in two stages using a hybrid QM/MM model and position restraints on the reactants, as implemented in the sander MD engine of the AMBER 18 software package.^37^ In the first stage of equilibration, the solvent of each system was equilibrated for 25 ps at constant volume and temperature (20 °C). In the second stage, the solvent was equilibrated for 1 ns at constant pressure (1 atm) and temperature (20 °C).

Following equilibration, conventional simulations of each system were run for 6 ns at constant temperature (20 °C) and pressure (1 atm) to sample different relative orientations of the unassociated azide and cation molecules, restraining the distance between the nitrogens of the azide and the central carbon of the cation to 20 Å. Temperature was maintained using a weak Langevin thermostat with a collision frequency of 0.001 ps^–1^, and pressure was maintained using the Monte Carlo barostat^38^ with a coupling constant of 1 ps^–1^. A time step of 1 fs was used. For each chemical reaction, an ensemble of 50 unassociated reactant conformations with diverse anion–cation orientations (Figure S2) was generated by saving conformations every 100 ps from the last 5 ns of the standard simulation. This ensemble of unassociated conformations was used to initiate WE simulations of the corresponding chemical reaction as described below.

### Weighted Ensemble Simulations of Reaction Pathways

The WE strategy, illustrated in Figure S2, involves initiating multiple weighted trajectories in parallel and iteratively applying a resampling procedure after propagating dynamics for a fixed resampling time interval τ.^12,13^ Configurational space is typically divided into bins along a progress coordinate. The resampling procedure aims to provide even coverage of configurational space with a target number of *N* trajectories per bin by either replicating trajectories that have made transitions to less-visited regions or terminating trajectories that have not made such transitions. Importantly, trajectory weights are tracked rigorously such that *no bias is introduced into the dynamics*, enabling direct estimation of rate constants. To maintain nonequilibrium, steady-state conditions, each trajectory that reaches the target state is “recycled”, terminating the trajectory and spawning off a new trajectory from the initial state with the same trajectory weight.

To generate a large ensemble of continuous pathways for each azide-clock reaction, we ran five independent WE simulations of the reaction using the open-source, highly scalable WESTPA 2.0 software package^39^ according to best practices.^40^ We initiated each WE simulation by randomly selecting five of the 50 unassociated reactant conformations sampled by conventional simulations and applied a resampling time interval τ (WE iteration) of 0.5 ps with a target number of 5 trajectories/bin. We used a two-dimensional WE progress coordinate consisting of the minimum separation distance between any nitrogen of the azide anion and (i) the central carbon of the cation and (ii) any carbon of the cation. This progress coordinate was binned as illustrated in Figure S3. Trajectories were recycled when the minimum separation distance between the azide and cation was <1.6 Å.

For each reaction, simulation convergence was assessed by examining the time-evolution of the overall reaction rate constant (*k*_rxn_), averaged over all five WE simulations (Figure S4). Each simulation was run for 500 WE iterations of applying the resampling procedure. As an additional verification of convergence, we first applied the weighted ensemble steady-state (WESS) reweighting procedure^41^ to each WE simulation after 500 WE iterations to reweight trajectories for a steady state and then restarted each WE simulation with the updated trajectory weights for an additional 100 WE iterations to ensure that the average rate constant remained at a steady value. Reweighting using the WESS procedure was performed using 75% of the preceding simulation data.

Dynamics of the WE simulations were propagated using the AMBER 2018 dynamics engine.^37^ Since the WE strategy requires the use of stochastic dynamics—ensuring that the dynamics of replicated trajectories diverge—we used a stochastic thermostat (i.e., a weak Langevin thermostat with a collision frequency of 0.001 ps^–1^) to maintain a temperature of 20 °C. A Monte Carlo barostat^38^ with a coupling constant of 1 ps^–1^ was used to maintain a pressure of 1 atm.

### State Definitions

For all analysis, definitions of key states were defined as follows (see also Figure S3). The unassociated reactants state was defined as a minimum separation distance between azide and cation of >10 Å. The target product state was defined as a minimum separation distance of <1.6 Å (as determined by RI-MP2 geometry optimizations of the products; see Figure S1). In addition, an ion-pair intermediate state was observed at minimum azide–cation separation distances between 5 and 2.25 Å.

### Calculation of Rate Constants

Rate constants are reported as averages based on five WE simulations along with 95% credibility regions using a Bayesian bootstrapping approach.^42^

For each WE simulation, unimolecular rate constants *k*_AB_ (i.e., *k*_*–*1_ and *k*_2_) for a transition from state A to state B were calculated using the Hill relation as follows:
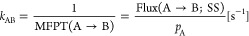
1where the inverse of the mean first passage time MFPT(A → B) for the A to B nonequilibrium steady state is equal to the conditional steady-state probability flux Flux(A → B; SS) into the target state B for trajectories most recently in the initial state A. To focus on the unidirectional flux in the forward direction of the reaction, the Flux(A → B; SS) was normalized by the steady-state population *p*_A_ in state A (i.e., sum of statistical weights of trajectories most recently in state A). Both the Flux(A → B; SS) and *p*_A_ were calculated as running averages over the WE simulation.

For bimolecular rate constants (i.e., *k*_rxn_ and *k*_*–*1_), the conditional flux was divided by the effective concentration of reactants *C*_0_ to yield rate constants in units of M^–1^ s^–1^.
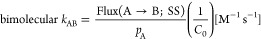
2Effective concentrations *C*_0_ for each simulation system (3.70, 3.75, and 3.69 mM for the 4-OCH_3_T^+^, T^+^, and 4-CF_3_T^+^ cations, respectively) were calculated as  where *N*_A_ is Avogadro’s number and *V* is the volume of the simulation box.

### Calculation of Percent Productive Collisions

Percent productive collisions for each reaction are reported as averages based on five WE simulations along with 95% credibility regions using a Bayesian bootstrapping approach.^42^ These percentages were calculated according to the following equation:
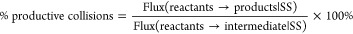
3where the numerator is the steady-state flux from the reactants to the products state and the denominator is the steady-state flux from the reactants to an ion-pair intermediate, which is defined as a distance of <5 Å between the two atoms of the reactant molecules.

### Calculation of Addition-Site Ratios

Ratios of azide addition at various sites on the cation were calculated directly from successful reaction pathway ensembles and reported relative to the addition site with the highest amount of flux (Figure S5). Since each of the three para-positioned addition sites for the T^+^ cation are symmetrically equivalent, the calculated ratio for those cations is the average of the total flux corresponding to addition at all three of these sites (likewise for the symmetrically equivalent sites of the 4-OCH_3_T^+^ and 4-CF_3_T^+^ cations).

### Clustering of Pathways into Distinct Classes

To cluster reaction pathways into distinct classes, we applied our recently developed linguistics pathway analysis of trajectories with hierarchical clustering (LPATH) method.^43^ This method involves the three steps detailed below.

In step 1, we discretized each pathway by assigning a state label to the configuration after each resampling time interval τ of 0.5 ps. Here, the state label is the symmetry-adapted identity of the phenyl-ring carbon atom that is nearest the nitrogen of the azide anion. For the T^+^ cation, this gives three states: T (center carbon), O (ortho or 2-carbon), and P (para or 4-carbon). For the 4-OCH_3_T^+^ and 4-CF_3_T^+^ cations, this gave five states: T (center carbon), O (ortho or 2-carbon at rings not containing the substrate), P (para or 4-carbon at rings not containing the substrate), X (ortho or 2-carbons at the ring containing the substrate), and S (the carbon to which the substrate is directly bonded).

In step 2, we quantified the similarity of each pair of discretized path sequences using a modified version of the Gestalt pattern matching algorithm,^44^ which is used in computational linguistics for comparing text strings of varying lengths in the detection of plagiarism. Using this algorithm, a distance between a pair of text strings was calculated using the following equation, which contains a correction factor in the denominator to account for pairwise pathway comparisons in which the pathway lengths are different from each other.
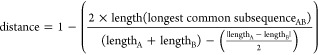
4

Finally, in step 3, we clustered the discretized path sequences into distinct pathway classes using the pairwise distances and a combination of a hierarchical agglomerative clustering algorithm and the Ward linkage method, which minimizes the variance within a given cluster.^45^ Based on the resulting tree diagram (dendrogram) of clusters, we identified distinct classes of pathways by positioning a horizontal line that maximizes the distance separation between nodes in the dendrogram (Figure S6).

## Results and Discussion

To enable direct simulations of complete pathways for each of the three azide-clock reactions, we applied the WE strategy with a hybrid QM/MM model in which the reactants were treated using quantum mechanics and the acetonitrile–water explicit solvent using molecular mechanics. For each reaction, five independent WE simulations were run, generating, in aggregate, thousands of pathways (1805, 8704, and 19,220 pathways for the 4-OCH_3_T^+^, T^+^, and 4-CF_3_T^+^ cations, respectively). The total conformations sampled from our simulations (every 0.5 ps) were 5.86 × 10^5^ for the 4-OCH_3_T^+^ reaction, 6.28 × 10^5^ for the T^+^ reaction, and 5.76 × 10^5^ for the 4-CF_3_T^+^ reaction. Each WE simulation was completed within 4 days using 280 Intel Xeon 2.6 GHz CPU cores in parallel.

### Simulations Reveal a Two-Step Mechanism

**Figure 2:**
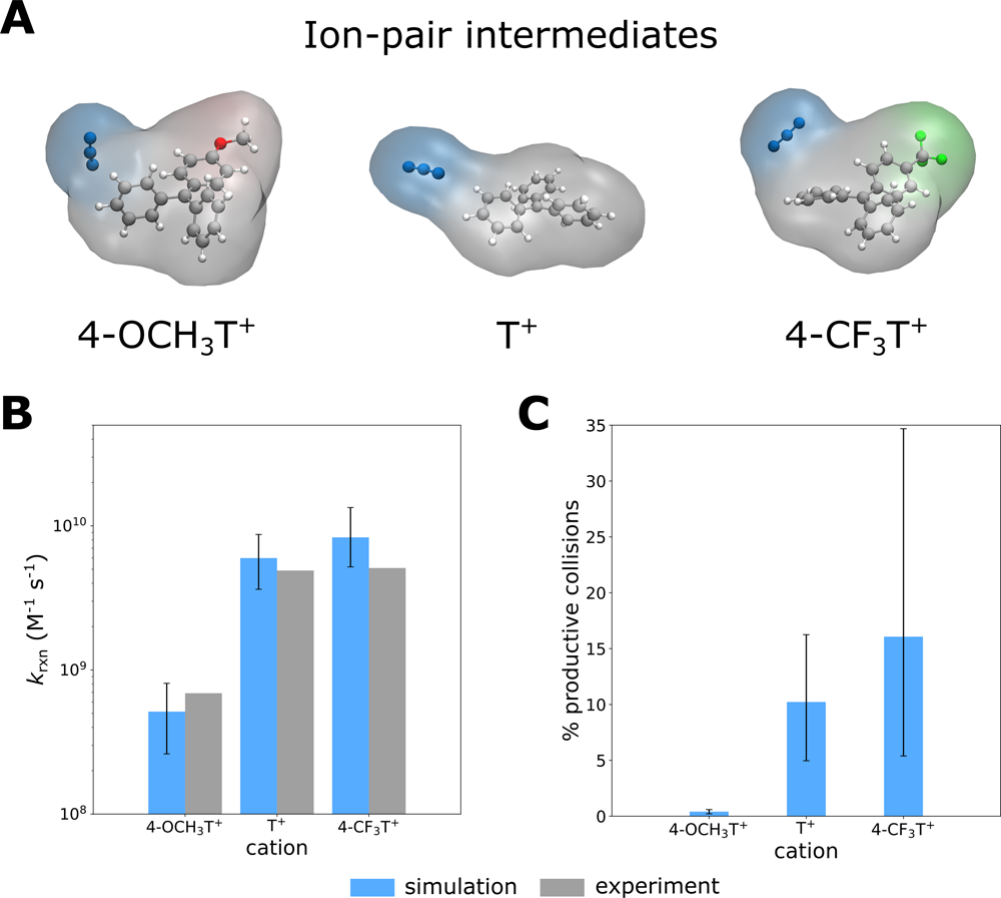
Direct simulations of reactions reveal structures of ion-pair intermediates and yield rate constants within error of experiment. (A) Representative ion-pair intermediate structures from the most probable reaction pathways. (B) Comparison of reaction rate constants calculated from simulation (blue) to those measured by laser-flash photolysis experiments^27^ (gray). (C) Percent productive collisions calculated from simulation follow the trend in reaction rate constants for azide addition to each of the three cations. Reaction rate constants and percent productive collisions are averages from five independent WE simulations with uncertainties that each represent 95% credibility regions, as estimated using a Bayesian bootstrap approach (see Methods).

Our simulations of each azide-clock reaction reveal a two-step reaction mechanism. In the first step, diffusional collision of the azide anion and trityl cation forms an ion-pair intermediate in which the anion and cation are within van der Waals contact, but not forming the target N–C bond with the central carbon of the cation. In the second step, the ion-pair intermediate “activates”, rearranging to the product. For each of the three reactions, the ion-pair intermediate involves the azide anion contacting a carbon of an unsubstituted phenyl-ring propeller (Figure 2A).

For each reaction, our calculated rate constant *k*_rxn_ for the overall reaction is within range of that measured by laser-flash photolysis experiments, reproducing the trend in reactivity of 4-OCH_3_T^+^ < T^+^ < 4-CF_3_T^+^ (Figure 2B). Consistent with this trend, the percentage of productive collisions (collisions of reactants that successfully reached the product state) is lowest for the least reactive 4-OCH_3_T^+^ cation (0.41 ± [0.21, 0.59]%) and higher for the more reactive 4-CF_3_T^+^ (16.07 ± [5.39, 34.69]%) and T^+^ cations (10.22 ± [4.96, 16.25]%) (Figure 2C). The substantially larger uncertainty in the percent productive collisions for azide addition to the 4-CF_3_T^+^ is due to one of the five WE simulations yielding a particularly high percentage.

### Reactions Are Activation-Limited

**Figure 3:**
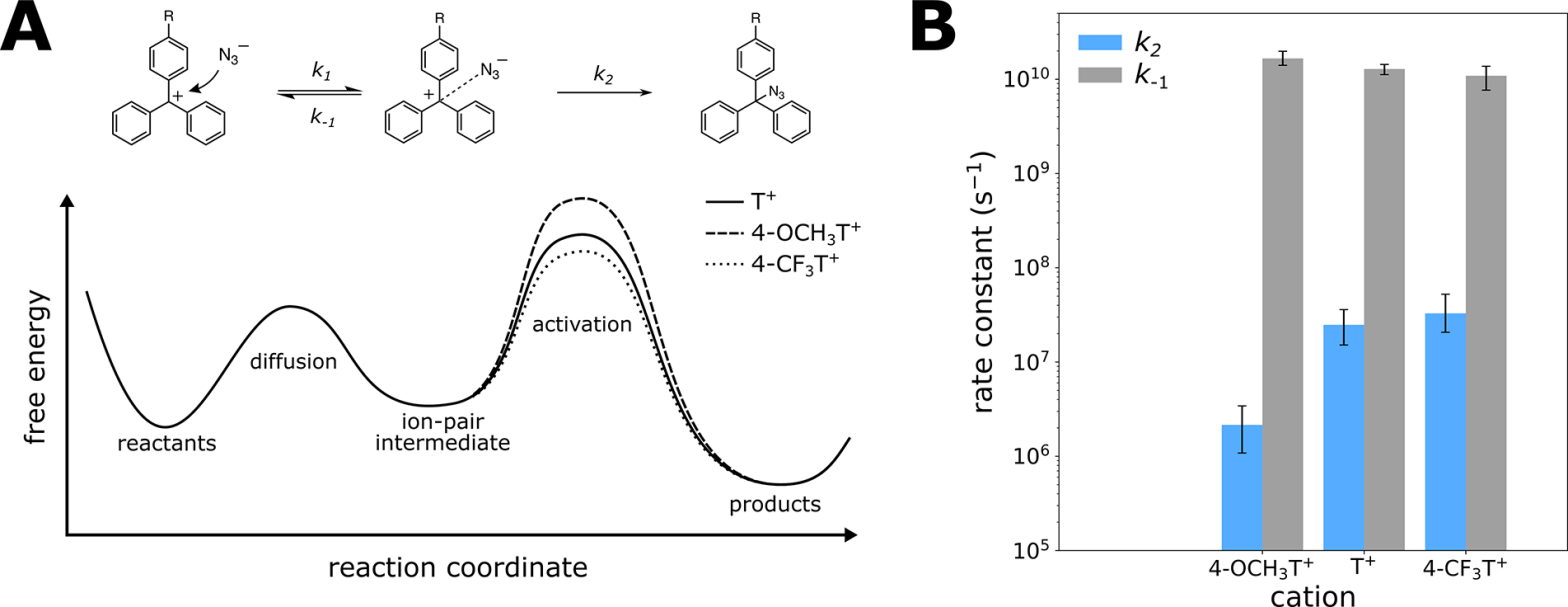
Evidence of activation-controlled reactions. (A) Schematic of the free energy profile for the azide-clock reaction as a two-step reaction involving the formation of an ion-pair intermediate and a rate-limiting rearrangement of this intermediate to the product. (B) Direct calculations of rate constants for the dissociation of the ion-pair intermediate (*k*_*–*1_) and rearrangement of the intermediate to the product (*k*_2_) reveal that for all three cations, *k*_*–*1_ ≫ *k*_2_, which indicates that the *k*_2_ step is rate-limiting (activation-controlled). The trend in *k*_2_ values explains the trend in the overall reaction rate constant *k*_rxn_, whereas *k*_1_ values are the same for all three reactions.

To determine the rate-limiting step for each reaction, we directly calculated rate constants for each individual step of the reaction (Figure 3A) from our simulations, i.e., *k*_1_ for formation of an ion-pair intermediate, *k*_*–*1_ for the dissociation of the intermediate, and *k*_2_ for the rearrangement of the intermediate to product (see Methods for state definitions). While the *k*_1_ values for all three reactions are essentially identical, the corresponding *k*_2_ values follow the trend in the overall reaction rate constant *k*_rxn_ (Figure 3B, Table S1) Due to the much more rapid dissociation of the ion-pair intermediate relative to rearrangement of the intermediate to the product (*k*_*–*1_ ≫ *k*_2_), the *k*_2_ step is rate-limiting for all three reactions. Thus, all three of the reactions in the present study are activation-limited rather than diffusion-limited. In contrast, previous fitting of data from flash photolysis experiments to a two-step mechanism suggested the activation step to be rate-limiting for the less reactive cations (e.g., 4-OCH_3_T^+^) and the diffusion step to be rate-limiting for the more reactive cations (e.g., T^+^, 4-CF_3_T^+^).^27^ However, these experiments monitored only the lifetime of the cation reactant, underscoring the value of using simulations to directly calculate rate constants for individual steps of a chemical reaction.

### The Activation Step Involves “Propeller Crawling”

A hallmark of WE simulations is not only the direct calculation of rates but also ensembles of continuous, unbiased pathways between the initial and target states (here, the reactants and products, respectively). While each WE simulation consists of many pathways with shared history (common trajectory segments), we were able to obtain two distinct classes of pathways for each reaction and their corresponding probabilities by clustering the pathways from all five WE simulations of that reaction based on the sequence of configurations visited (see Methods).

**Figure 4:**
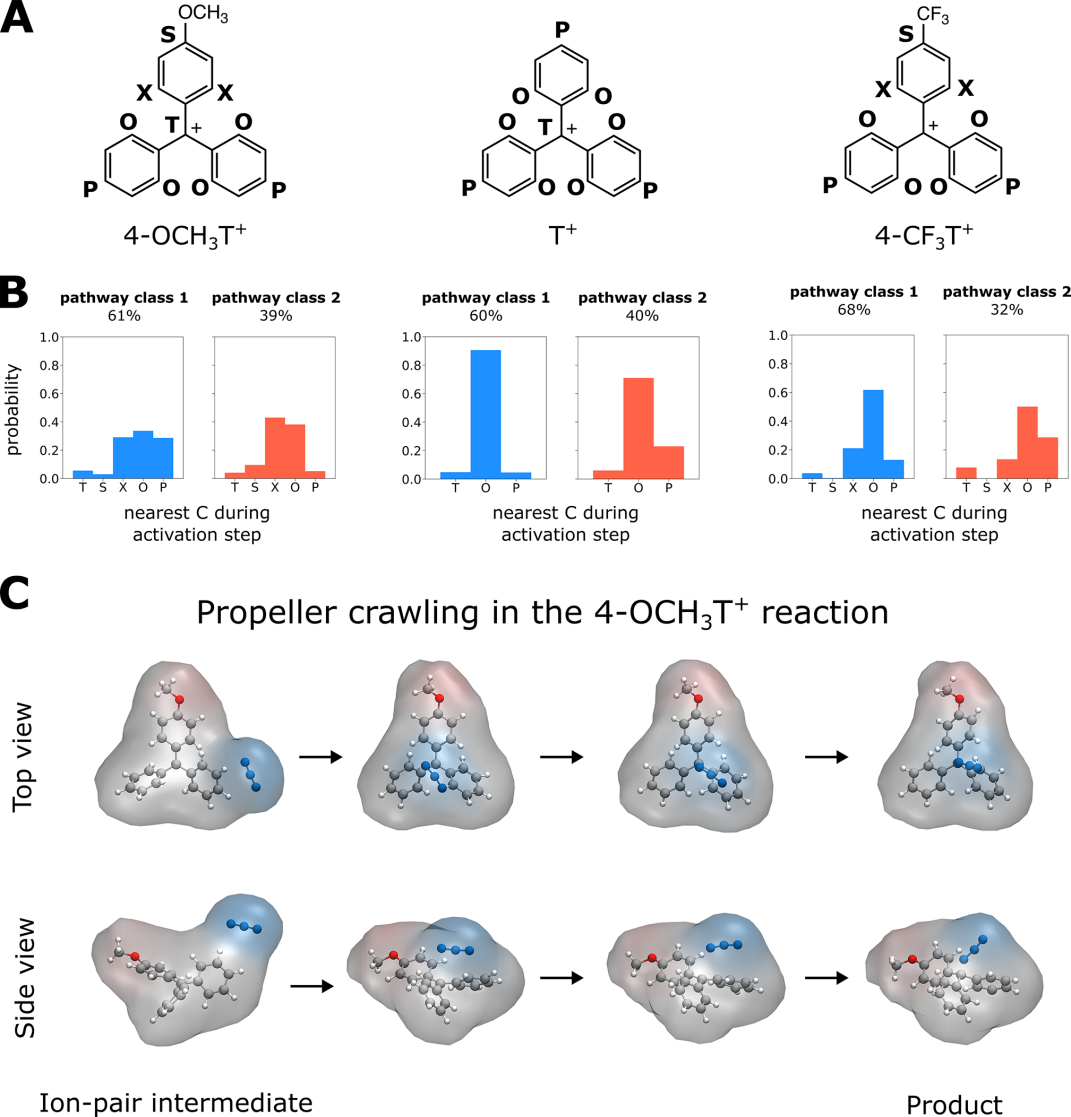
Distinct classes of pathways reveal differences in mechanism among the three reactions. (A) Symmetry-adapted labels for carbons of the phenyl rings that are contacted by the azide anion during its crawling to the target central carbon (T) of the cation: S is the carbon bonded to the substituent of a substituted phenyl ring, X is the ortho-positioned carbon that is bonded to the substituent, O is the ortho-positioned carbon of an unsubstituted phenyl ring, and P is the para-positioned carbon of an unsubstituted ring. (B) Probability distributions as a function of carbon atoms contacted by the azide anion at any time during the activation step, as considered every 0.25 ps. The azide displays more even crawling across all carbons considered in the 4-OCH_3_T^+^ cation. (C) Snapshot configurations of the propeller-crawling mechanism during the activation step along the most probable reaction pathway for the 4-OCH_3_T^+^ cation.

Our pathway analysis reveals that the activation step involves the azide anion “crawling” among the cation’s three propellers (phenyl rings) on its journey to the cation’s central carbon. Further, the trend in the rate constants *k*_2_ for the activation step of 4-OCH_3_T^+^ < T^+^ < 4-CF_3_T^+^ (Figure 3B) is due to the 4-OCH_3_T^+^ reaction involving a greater range of “propeller crawling” relative to the T^+^ and 4-CF_3_T^+^ reactions; that is, the azide anion is more likely to contact the ortho- (X) and para-positioned (S) carbons of the cation’s propellers before reaching the target central carbon (T) of the cation to form the target N–C bond (Figure 4). This greater probability is likely due to the reduced partial positive charge of the 4-OCH_3_T^+^ cation’s central carbon relative to T^+^ and 4-CF_3_T^+^. Movies of this propeller-crawling mechanism are provided for the most probable pathway of each reaction (Movies S1–S3); in addition, Figure 4C shows snapshots from the activation step of the most probable reaction pathway for the 4-OCH_3_T^+^ cation, which exhibits the greatest extent of crawling.

A closer examination of the propeller-crawling mechanism reveals interesting features for each reaction. For the unsubstituted cation, the most probable pathway class involves a greater range of propeller-crawling compared to the minor pathway class with a higher probability of the azide anion first contacting an ortho-positioned carbon of a phenyl ring to form the ion-pair intermediate followed by contacts with both the ortho and para positions of phenyl rings while crawling to the central carbon of the cation. For the 4-OCH_3_T^+^ cation, the minor pathway class (class 2) involves a high probability of the azide anion first contacting the carbon that is directly bound to the methoxy substituent; this carbon is more positively charged compared to the other carbons in the phenyl ring due to resonance effects of the electron-donating methoxy substituent. For the 4-CF_3_T^+^ reaction, azide addition did not occur at the S-labeled carbon in Figure 4A, which is directly bound to the trifluoro substituent.

### Detection of Azide Addition at Propeller Sites

Our reaction simulations captured azide addition at not just the target central carbon of each cation but also at the 2- or 4-positions of the cation’s phenyl-ring “propellers” (Figure S5). These propeller sites of addition would be expected based on the delocalization of positive charge in resonance models of the cations; however, the corresponding addition species result in nonaromatic rings and would therefore be too transient for detection in the previously reported flash photolysis experiments.^27^ These species likely rearrange to the expected product (involving azide addition to the cation’s central carbon) to preserve aromaticity of the phenyl rings. While such rearrangements are beyond the target states of our simulations, their occurrence would further contribute to the activation step being rate-limiting for the reactions.

### Outlook for Simulating Chemical Reactions

We have demonstrated the power of the WE strategy in enabling the direct molecular simulation of pathways and rate constants for chemical reactions using hybrid QM/MM models. Such simulations would be feasible for chemical reactions on the μs time scale with <50 reacting atoms in the QM region using a semiempirical level of theory. The reactions can be either in solution or enzyme-catalyzed. The future looks bright for simulating chemical reactions with more complex reactants and/or higher levels of QM theory, including the use of GPU-accelerated dynamics engines that can greatly extend the time scales of hybrid QM/MM simulations (NAMD)^46^ and deep-learning potentials approaching the “gold standard” of coupled-cluster accuracy at the cost of a classical force field (ANI-1ccx).^47^

## Conclusions

We report direct molecular dynamics simulations of azide-clock reactions in explicit solvent involving the addition of an azide anion to each of three different cations, 4-OCH_3_T^+^, T^+^, and 4-CF_3_T^+^. These simulations were enabled by applying the WE path sampling strategy with hybrid QM/MM models. Our simulations generated thousands of continuous pathways for each reaction, yielding reaction rate constants that are within error of experiment. Results revealed that each reaction involves a two-step mechanism in which the first step involves diffusional collision of reactants to form an ion-pair intermediate, and the second step involves “activation” or rearrangement of the intermediate to the product. In contrast to previous assumptions, all three reactions are activation-controlled rather than diffusion-controlled. Based on our simulated ensemble of reaction pathways, the slower activation step of the 4-OCH_3_T^+^ reaction relative to the T^+^ and 4-CF_3_T^+^ reactions is due to the ability of the azide to “crawl” along a greater range of the cation’s three propellers (phenyl rings). This “propeller-crawling” mechanism underscores the importance of dynamical effects on this series of azide-clock reactions. Our work not only provides the most detailed views to date of these reactions but also presents a rare-events sampling method that enables simulation of pathways and kinetics for many chemical reactions, either in solution or enzyme-catalyzed.

## Data Availability

Initial coordinates, parameters, and input files for weighted ensemble simulations of each reaction can be found on GitHub: https://github.com/westpa/user_submitted_scripts/tree/main/chemical_reactions_we.

## Supporting Information Available

Geometries of reactants and products at the PM6-D and RI-MP2 levels of theory; an overall workflow of the WE strategy; binning scheme and state definitions used for WE simulations and analysis; time-evolution of rate-constant estimates from WE simulations for each reaction; dendrograms used to determine distinct pathway classes; ratios of probabilities for azide addition to propeller carbons; a table containing all computed rate constants (PDF)
Movie of the most probable reaction pathway for the 4-OCH_3_T^+^, 4-CF_3_T^+^ and T^+^ chemical reactions (MP4)
(MP4)
(MP4)
