# Supplementary material for: Revisiting Textbook Azide-Clock Reactions: A “Propeller-Crawling” Mechanism Explains Differences in Rates
Source: J Am Chem Soc. 2024 Apr 30;146(18):12828–35. doi: 10.1021/jacs.4c03360 (PMC11078601; doi:10.1021/jacs.4c03360)
Supplement: Supplementary file 1 — ja4c03360_si_001.pdf [file ja4c03360_si_001.pdf]

Revisiting textbook azide-clock reactions: A "propeller-crawling" mechanism explains differences in rates

Anthony T. Bogetti,[†] Matthew C. Zwier,[‡] and Lillian T. Chong[*,†]

†*Department of Chemistry, University of Pittsburgh, Pittsburgh, Pennsylvania 15260, United States*

‡*Department of Chemistry, Drake University, Des Moines, Iowa 50311, United States*

E-mail: ltchong@pitt.edu

Supporting Information

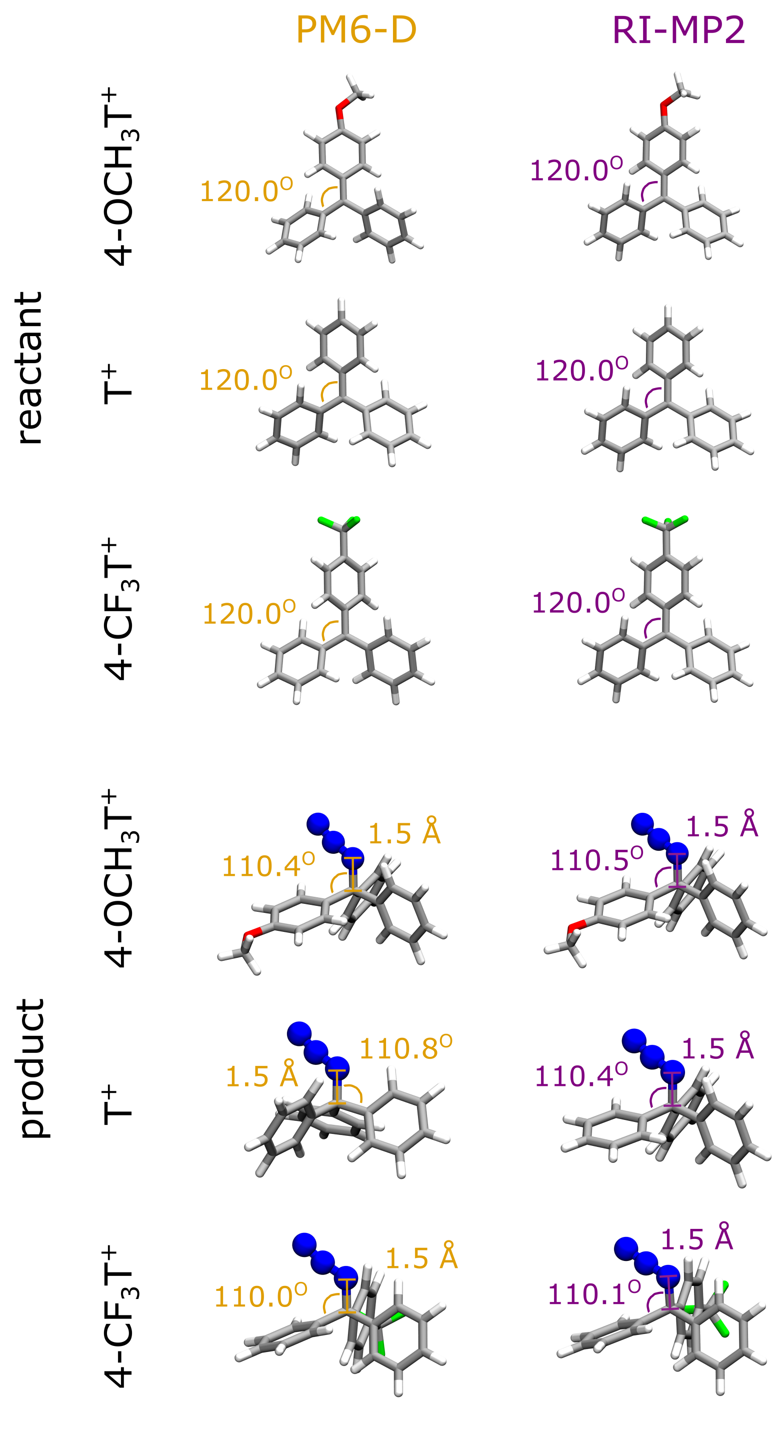

Figure S1: Optimized geometries of the reactant cation and product for each reaction at the PM6-D and RI-MP2 levels of theory. As mentioned in Methods, we initiated all simulations from RI-MP2 optimized geometries. Geometry-optimized structures using the PM6-D level of theory resemble those from a higher level of theory (RI-MP2) for reactants and products.

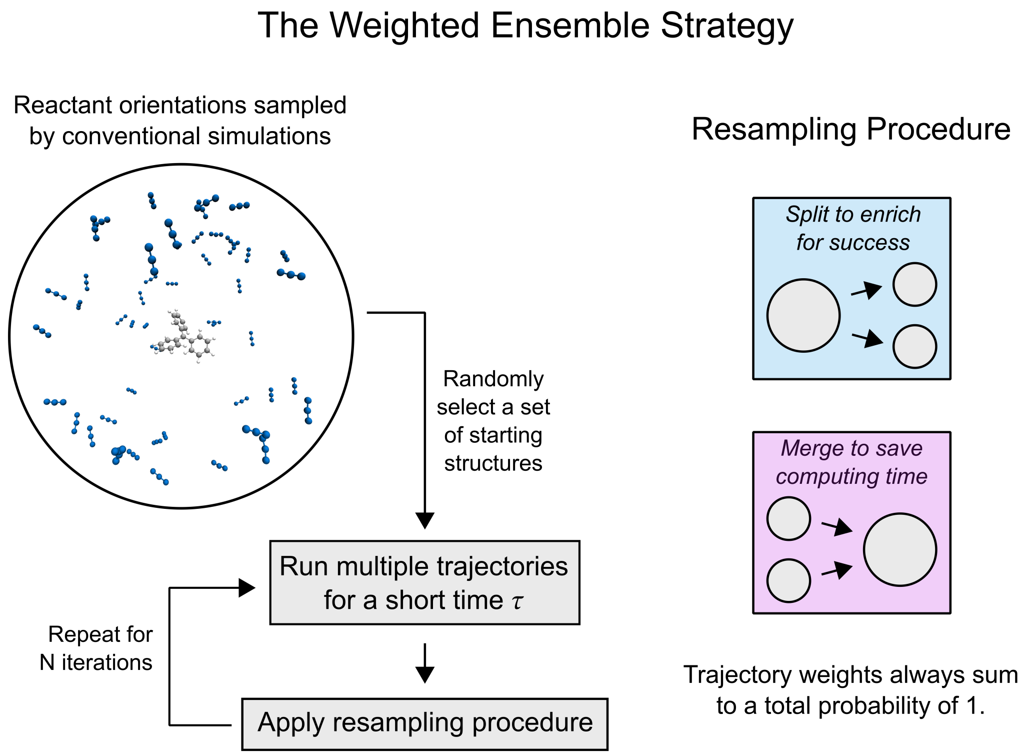

Figure S2: Overall workflow of the weighted ensemble (WE) strategy. The weighted ensemble (WE) strategy involves running multiple trajectories in parallel and iteratively evaluating trajectories for resampling after a short time interval τ. Configurational space is divided into bins along a progress coordinate and the goal of the resampling procedure is to split or merge trajectories to yield a target number of trajectories per bin. Trajectory weights (indicated by circle sizes) are rigorously tracked such that no statistical bias is introduced in the dynamics, enabling direct calculation of rates from the simulation. For each of the reactions in this study, five WE simulations were initialized from five structures randomly selected from a set of 50 unassociated-reactant configurations sampled by a conventional simulation and run for N = 500 iterations with a two-dimensional progress coordinate, τ = 0.5 ps, and a target number of 5 trajectories/bin to generate a sufficient number of pathways to yield statistically robust rates (see Methods).

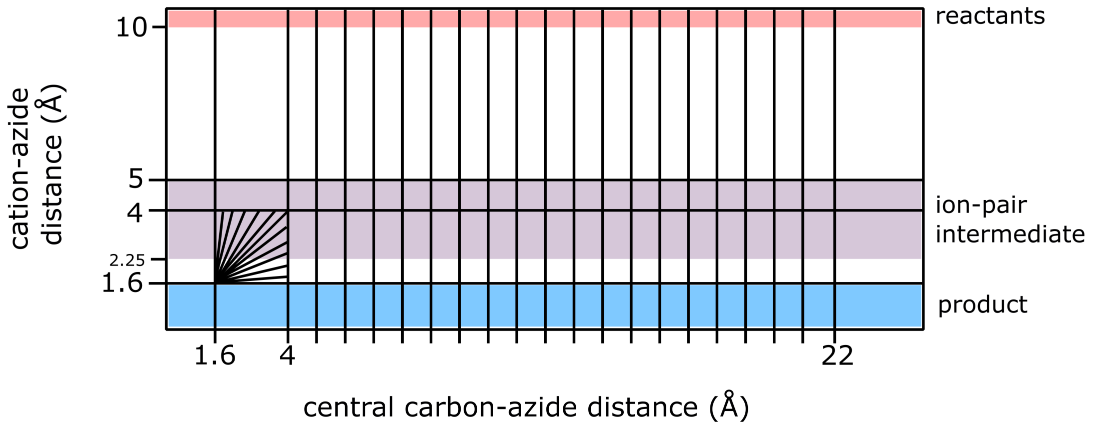

Figure S3: Progress coordinate, state definitions, and binning scheme used for WE simulations of the azide-clock reactions. As mentioned in Methods, we used a two-dimensional progress coordinate consisting of the minimum separation distance between any nitrogen of the azide anion and (i) the central carbon of the cation, and (ii) any carbon of the cation. Shaded regions indicate state definitions for the reactants (pink), ion-pair intermediate (purple), and product (blue). Bins were positioned every 1 Å from 4 Å to 22 Å along the first dimension and from 4 Å to 5 Å in the second dimension. To focus sampling on the rate-limiting activation step involving rearrangement of the ion-pair intermediate to the product, a radial binning scheme was used for the region between 1.6 Å and 4 Å in both dimensions, positioning radial bins from (1.6 Å, 1.6 Å) to (4 Å, 4 Å) at 1° intervals.

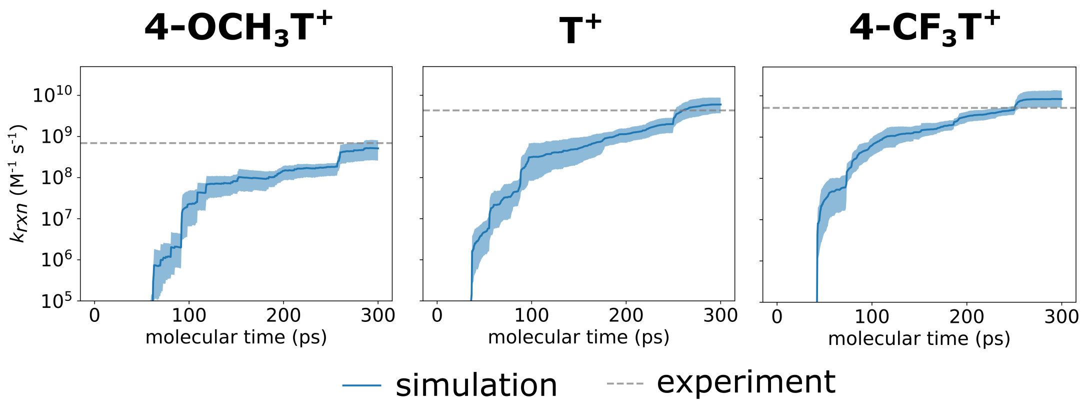

Figure S4: Time evolution of k_{rxn} for azide addition to each cation. A cumulative average of k_{rxn} for each reaction shows that the rate constants begin to level off after 250 ps (500 WE iterations). The jump in the rate-constant estimate at 251 ps (WE iteration 501) is due to reweighting for a steady state using the WESS procedure. After reweighting, 50 ps (100 WE iterations) of additional simulation reveals that the rate constant average levels off further, suggesting our estimates are reaching convergence.

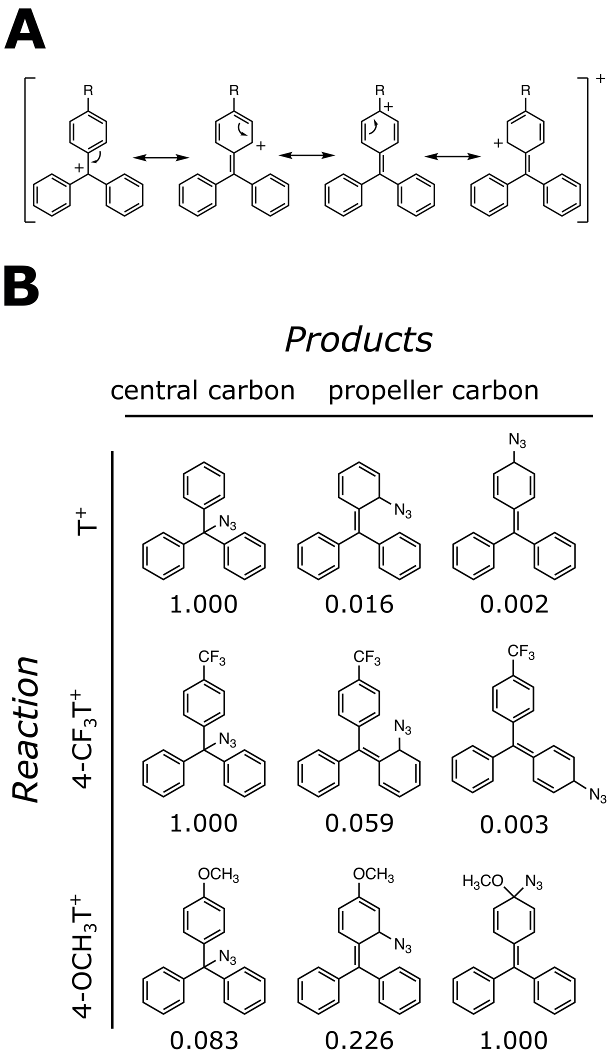

Figure S5: (A) Resonance models predict the dominant product to involve addition to the central carbon atom of the cation. Addition to various phenyl "propeller" carbons is possible, but would form an unstable product with a nonaromatic ring. (B) Ratios of probabilities for azide addition to propeller carbons relative to addition to the central carbon of the cation calculated from WE simulation.

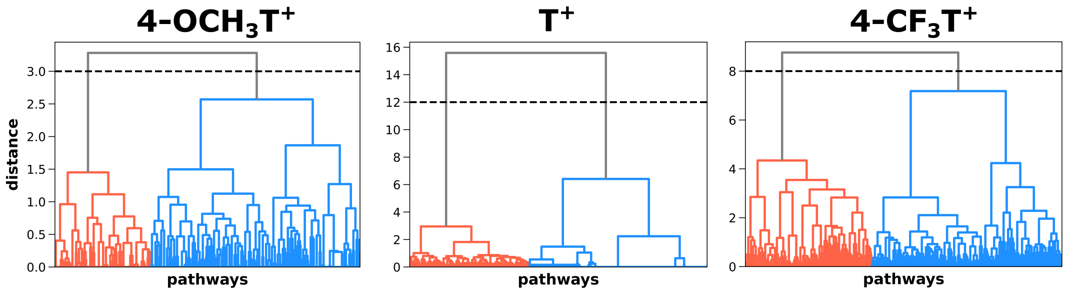

Figure S6: Each dendrogram (tree diagram) reveals clusters of pathways that are more related to each other than to other pathways in terms of sequence of configurations visited (see Methods). To obtain distinct classes of pathways, a horizontal line (dashed line) was positioned to maximize the distance separation between nodes in the dendrogram.

Table S1: Computed rate constants for azide addition to each of the three cations. Rate constants were directly computed from WE simulations and reported as averages from five independent WE simulations with uncertainties that each represent the 95% credibility region from Bayesian Bootstrapping.[1] Overall reaction rate constants (k_{rxn}) from experiment were previously measured by others using laser-flash photolysis.[2]

	experiment	simulation				
cation	k_{rxn}x10^9 ($M^{-1}s^{-1}$)	k_{rxn}x10^9 ($M^{-1}s^{-1}$)	k_1x10^{11} ($M^{-1}s^{-1}$)	k_{-1}x10^{10} (s^{-1})	k_2x10^7 (s^{-1})	% productive collisions
4-OCH_3-T^+	0.69	0.51 [0.26,0.81]	1.77 [1.14,2.61]	1.66 [1.41,1.98]	0.22 [0.11,0.34]	0.41 [0.21,0.59]
T^+	4.90	5.98 [3.63,8.72]	1.25 [1.09,1.47]	1.29 [1.12,1.44]	2.50 [1.50,3.60]	10.22 [4.96,16.25]
4-CF_3-T^+	5.10	8.33 [5.19,13.43]	1.12 [0.97,1.26]	1.09 [0.77,1.38]	3.30 [2.10,5.20]	16.07 [5.39,34.69]

Movies S1-S3: Movies of the most probable reaction pathways for addition of azide anion to the (S1) 4-OCH_3-T^+, (S2) T^+, and (S3) 4-CF_3-T^+ cations. These movies reveal a greater range of azide crawling among the three phenyl-ring "propellers" of the cation for the less-reactive 4-OCH_3-T^+ cation relative to the T^+ and 4-CF_3-T^+ cations.

References

(1) Mostofian, B.; Zuckerman, D. M. Statistical Uncertainty Analysis for Small-Sample, High Log-Variance Data: Cautions for Bootstrapping and Bayesian Bootstrapping. *J. Chem. Theory Comput.* **2019**, *15*, 3499–3509.

(2) McClelland, R. A.; Kanagasabapathy, V. M.; Banait, N. S.; Steenken, S. Reactivities of Triarylmethyl and Diarylmethyl Cations with Azide Ion Investigated by Laser Flash Photolysis. Diffusion-controlled Reactions. *J. Am. Chem. Soc.* **1991**, *113*, 1009–1014.
